# The Institutional Library

**Published:** 1918-02-02

**Authors:** 


### THE INSTITUTIONAL LIBRARY.

**Handbook of Physiology.** By W. D. Halliburton, M.D., LL.D., F.R.S. Thirteenth Edition. (London: John Murray. 1917. Pp. 930 + xix. Price 16s. net.)

This is a book which can boast a great tradition. As Kirke's "Physiology" it claimed thirteen editions, and under Dr. Halliburton's guidance it has now lived into another twelve. In such circumstances the voice of the reviewer is surely a superfluity, for a stronger note than any he can utter has already been sounded. We cannot enumerate all the numerous excellences of the volume, but we wish cordially to recognise them. Good teaching in physiology is of the highest importance to the student of medicine, for it is one of the foundations of his clinical work, and in providing such teaching Dr. Halliburton has done excellent service.

We may say a single word on the special relation of the book to the medical student. There is always a difficulty in adjusting the respective claims of the preliminary and practical parts of the medical curriculum. Possibly the pure physiologist may object to his subject being placed in the preliminary class. Yet for the physician and surgeon to be, this is its true position. It has no doubt wider claims, but here it is a means to an end, and it must be taught with this end in view. If the practical physician is apt to over-emphasise this claim, not less true is it that the scientific physiologist is inclined to forget it. In his first chapter Dr. Halliburton recognises the claim and intimates an endeavour to meet it. We are not certain that in this respect he has fully utilised his opportunities. Thus it will, we fancy, be agreed that the effect of lesions in different parts of the visual tract on the characters of the visual fields conveys both an impressive physiological

### THE INSTITUTIONAL LIBRARY—(*continued*).

lesson and aids the student in the clinical problems of cerebral localisation. To judge from a personal experience, the student rarely comes to the hospital wards provided with this equipment; and this is not surprising, for his physiological text-books do not supply it. In this respect Dr. Halliburton is not more helpful than his peers. Take again the study of the circulation. Nowadays the polygraph is largely used for this purpose in clinical work, and its records convey important physiological teaching. Why should not such knowledge, with its scientific and practical values, be used in the training of the student? It speaks sound physiological doctrine, and is a necessary equipment in clinical work. But here we look for it in vain. Of course it may be said, and said quite truly, that a student's text-book cannot contain everything. We agree. But out of such a consideration arises the necessity for selection, and we venture to think that the polygraph and its tracings will give a better training in physiology, and enormously higher clinical values, than the story of half-a-dozen kymographs no one of which the student will ever see again once he has effected his escape from the physiological laboratory. Not one word have we to say against discipline in scientific method, but it is just as easy to illustrate principles by useful and serviceable knowledge as by knowledge which, for the medical student, can never have a field of application. Many of our text-books are very learned, and contain stores of information quite relevant to their professed aim. But they do not always remember that the student has many other subjects to compass, that the time at his disposal is limited, and that his main purpose in their perusal is to prepare himself for clinical work. Perhaps if we could secure the joint authorship of a physiologist and a physician the ideal medical text-book in physiology might be born. There is, however, a risk. The authors might quarrel. In the meantime, tried by many tests, Dr. Halliburton's volume justifies the success which it has so largely achieved.

**The Pasteurisation of Milk from the Practical View-Point.** By CHARLES H. KILBOURNE, late Chief of the Division of Pasteurising Plants, New York City Department of Health. (New York: John Wiley and Sons. London: Chapman and Hall, Ltd. 1916.)

THE book before us is a short practical manual dealing with the technique of milk pasteurisation. It contains a full description of the main varieties of apparatus used for this purpose, with diagrams, and should be of great use to all concerned in this branch of dairy industry. So far as we are aware the same information is not contained in any other book of such convenient form for practical reference, and hence the volume under review should find a considerable sphere of usefulness amongst inspectors and dairy technologists.

The pasteurisation of milk is more generally employed in America than in this country. Pasteurised milk in New York City Department of Health means milk that has been heated to from 142° to 145° F. for at least thirty minutes. By this process pathogenic bacteria are held to be destroyed, and the biochemical properties of the milk are held to be not sufficiently disturbed to alter seriously its nutritive value. Great care must be taken in the exact graduation of temperature, or results distinctly deleterious to the nutritive value of the milk will accrue, hence it is extremely important that those in charge of this part of hygienic dairying should have an intimate knowledge of the apparatus in use, and the principles involved; the book before us will certainly supply this. The economic division of labour which has brought about the phenomenon that, generally speaking, food must be produced in one area and consumed in another, has rendered it necessary to develop all methods which shall prevent either food waste or food contamination. The complicated manipulations which so perishable a food product as milk must necessarily undergo in its transit from the cow to the table render milk a very special object for the ingenuity of technical hygiene. The numerous regulations by which the consumer of milk is protected show the importance that the State attaches to a pure milk supply. As one of the aids to a pure milk supply pasteurisation takes a most prominent place, but it will not cover original dirty dairying and all the initial care in milking, filtering, and cooling, and the final care in ultimate delivery must be maintained if the full benefit is to be derived from it. We think the book before us is distinctly to be recommended, and great credit is due to the author for a clear exposition of a complicated subject.

**Lectures on Medicine: a Handbook for Nurses.** By Chalmers Watson, M.D., F.R.C.P.E. (Edinburgh: E. and S. Livingstone. 1917. Pp. 295+viii. Price 4s. 6d. net.)

DR. CHALMERS WATSON tells us in his Preface that "the nurse should have a more thorough training in medicine than is possible in a short course of lectures." Hence he has issued this book. It deals with various diseases according to the conventional text-book plan, discussing in each instance ætiology, diagnosis, prognosis, and treatment. In view of the size of the book these discussions are necessarily sketchy and superficial—the half-truth which is more dangerous than ignorance. Dr. Watson thinks that such an equipment will increase the efficiency of a nurse. For our own part we hold to the view that for the responsibilities involved in the diagnosis and treatment of disease nothing short of a complete medical curriculum will suffice, and to encourage untrained persons to dabble in them is an unhappy and mischievous enterprise. To teach nurses the art of nursing is an excellent ambition, but to lead them to believe that they ought to cultivate a smattering of medicine is dangerous alike to nurses and to the public. The amateur is bad enough in all conscience, but the semi-professional is *capable de tout*.

Judged by the standard the author selects the book has its merits, and the section dealing with the preparation of foods for invalids and convalescents is distinctly useful. For some reason or another the illustrations include a single pulse tracing; this is marked "pulsus alternans," but hardly merits such a description. Occasionally there is some carelessness in composition, as in the sentence "in many cases the best cardiac tonic we can prescribe is to reassure *the patient* that *their* condition is not serious, or, at least, not half as serious as *he* imagines." [Italics not in the original.] Apart from its syntax, who, we wonder, are included in the "we" of the principal clause? Does it include or exclude the audience?

**Electro-Therapeutics for Military Hospitals.** By WILFRID GARTON, M.R.C.S. (London: H. K. Lewis and Co., Ltd. 1917. Pp. 84 + viii. Price 2s. 6d. net.)

MR. GARTON has held a temporary commission in the R.A.M.C. for some two years, and during this time has had much experience with electrical methods of treat-

**THE INSTITUTIONAL LIBRARY**—(*continued*).

ment. He is convinced that these methods are not sufficiently used, and that their neglect means the delay in hospital of many patients who might otherwise be promptly rendered efficient. His book gives an account of the apparatus required, and of its employment as a therapeutic agent in various nervous and muscular lesions. The plan is carried out with much success, and the book will be helpful to any practitioner who desires to cultivate the methods with which it deals.

**The Venereal Diseases Problem.** By J. K. WATSON, M.D. (London: Baillière, Tindall and Cox. 1917. Pp. 54 + viii. Price 2s. 6d. net.)

THE main part of this book is occupied by a necessarily slight and superficial account of the symptoms, diagnosis, and treatment of the several forms of venereal disease, while in a final chapter appear some general considerations bearing on the social problem which these diseases create. The intention of the writer is to provide instruction for nurses and midwives. That some teaching is necessary for those engaged in nursing sufferers from venereal diseases may be allowed, and in some respects Dr. Watson's pages may be helpful for this purpose. But, in our view, he enters into unnecessary detail on diagnosis and treatment, and thus he would seem to encourage nurses to profess opinions and to undertake responsibilities which they ought to be warned specially to avoid. The best instruction for nurses is not provided by a sketchy and incomplete medical text-book. In his more general remarks Dr. Watson is more fortunate, and his last chapter provides information which might well be circulated among the general public.

**Outlines of Nursing History.** By MINNIE GOODNOW, R.N., formerly Directress of Nurses, Milwaukee County Hospital. Illustrated. (Philadelphia and London: W. B. Saunders Company. Price $2 net.)

THIS is a text-book history, useful to nurses for whom the history of their profession forms a subject of examination. After dealing briefly with nursing in ancient times and in the middle ages, a chapter on the Deaconess movement prepares the way for two upon the work of Florence Nightingale, and the establishment of trained nursing in Great Britain. The book then discusses nursing and the training-schools of America, including nursing in the Civil War. The training-schools of Europe and Asia are then considered, while the concluding chapters discuss nursing in the present war, existing nursing organisation and newspapers, and the opportunities for nurses in the diverse branches of their profession. Like most text-books, the present volume attempts to cover more ground than it can deal with adequately. The succession of short, titled paragraphs and the summary of points at the end of each chapter are more useful for examinees than inviting to the general reader. For those who require something more than a work of reference Sir Edward Cook's "Life of Florence Nightingale" makes far more fruitful reading, since Miss Nightingale's life and theories cover the whole ground of a nurse's profession, and show, in the drama of her own life, the difficulties, the ideals, and the practical application of principles which nursing involves for every woman who intends to be something more than a salaried official. But within the scope set to herself by the author "Outlines of Nursing History" can be recommended as a text-book which will be useful to those who wish to pass an examination in the history of their profession. The glazed pages, so popular in American publications, for the sake of the illustrations which they permit, are never pleasant to read or to handle; but these are the details which a text-book employs, and must be accepted accordingly.

**The Ideal Nurse.** AN ADDRESS TO NURSES. By CHARLES A. MERCIER, M.D., F.R.C.P., F.R.C.S., etc. (Mental Culture Enterprise, 329 High Holborn, W.C. 1. Price 1s. 3d. net.)

THIS brochure should be taken as a cordial by all who are suffering from the professional pessimist in his many manifestations. We commend to Colonel King-Harman, whose book we review below, Dr. Mercier's comment on the halcyon days of old: "It is scarcely possible for us to realise the degree of brutality that prevailed 100 years ago in all departments of life." In his retrospect of changes wrought through the inspiration of Christian charity, the writer lays stress on the improved treatment of the insane under the ægis of the trained nurse, and few, we believe, could read unmoved the fine pages in which he sums up the qualities and reward of his Ideal Nurse. Often as the reader may have pondered over the vocation of the nurse, and have striven to put into words the essence of good nursing, there is still much to learn from this wise physician, who has had his part in moulding the lives of many women to serve the highest ends, and who paints their crowning satisfaction in the recovery of a patient as "a joy as refined and as pure as that of the angels of heaven over the sinner that repenteth." An ideal gift to slip into a letter to a nurse.

**British Boys: Their Training and Prospects.** By COLONEL J. KING-HARMAN. (London: G. Bell and Sons. 1917. Price 2s. net.)

THIS book is written in the strain of exaggerated depreciation of all modern progress and ideas which was familiar enough before the war. In the light of present events it is not easy to read patiently an indictment of our whole nation for its supposed degeneracy and inferiority to a past very imperfectly visualised by the writer. Defects there are in plenty in our educational system, but after all that can be said remains the supreme test, "By their fruits ye shall know them." It would become us better to try to discover wherein lies the inherent excellence of the system which, in spite of obvious defects, has given us an army of heroes than to give vent to such cheap vapouring as that "the war has brought out the character which the schools had abstained from cultivating." In truth, the intolerant and indiscriminating dissatisfaction expressed by the writer with all that differs from his superficial impressions of the past can serve no useful purpose, and the few practical suggestions which he puts forward—as, for instance, the plea for medical inspection in the public schools and for improved teaching of science—convey no fresh information and are merely ejaculatory. A more thorough knowledge of English social conditions during the past hundred years or so would have saved the writer from ignorant rhapsodising over bygone abuses, and a little sympathetic study of modern educational methods would have taught him how many hundreds of earnest workers are giving their lives to remedy the very defects he believes himself to have discovered in the upbringing of British boys.

**Low's Handbook to the Charities of London, 1917.** (Sampson Low, Marston and Co., Ltd.) To anyone who does not seek detailed statistical information this is a useful list of the chief charities of London. The 1917 issue contains an illustrated account of St. George's Hospital, with a drawing of the proposed new building at Hyde Park Corner.

